# Citizen science provides insights on pollination services in urban community gardens

**DOI:** 10.1186/s12862-026-02507-x

**Published:** 2026-03-13

**Authors:** Susan Karlebowski, Monika Egerer, Astrid E. Neumann, Julia M. Schmack, Aaron N. Sexton, Ulrike Sturm

**Affiliations:** 1 Museum für Naturkunde Berlin - Leibniz Institute for Evolution and Biodiversity Science, Invalidenstraße 43, 10115 Berlin, Germany; 2 Urban Productive Ecosystems, Department of Life Science Systems, School of Life Sciences, Technical University of Munich, Hans Carl-von-Carlowitz-Platz 2, 85354 Freising, Germany; 3 Organismic and Cellular Interactions, Ludwig-Maximilians-University of Munich, Biocenter, Martinsried, Germany; 4 School of Integrative Plant Sciences, Cornell University, Ithaca, NY USA

**Keywords:** Fruit set, Crops, Bees, Urbanization, Urban agriculture, Ecosystem services, Mixed-methods, Participation types, Transdisciplinary research

## Abstract

**Background:**

Urban community gardens are collectively managed (agro)ecosystems shaped by diverse gardening practices that influence both planned and associated biodiversity. Yet, biodiversity-mediated ecosystem services in these gardens remain underexplored, and gardeners are rarely engaged as collaborators in scientific research. In this study, we investigated how bee diversity and urbanization factors affect pollination services, measured by the fruit set of common garden crops under real-world gardening conditions. Additionally, we examined participation dynamics within our citizen science approach and explored their relationship with the collected data as well as participants-reported challenges such as poor plant health.

**Results:**

We collaborated with 73 gardeners in 22 gardens in Berlin and Munich, Germany, to measure the fruit set of 150 crops, mainly from the families *Cucurbitaceae* and *Solanaceae*. In parallel, researchers conducted systematic bee observations and quantified urbanization factors such as air temperature and surrounding imperviousness. We found a significant positive interaction between bee species richness and landscape imperviousness for the fruit set of *Cucurbitaceae*, emphasizing the importance of species-rich bee communities for pollination services in urban gardens. In contrast, no significant environmental predictors explained the fruit set of the *Solanaceae* group. Instead, we found that differences in sampling frequency led to significant differences in fruit set measurements, suggesting that self-pollination or an observer bias may contribute to variability. Participation types (differing in duration and frequency of data collection) were not affected by plant health issues or difficulties with the protocol, indicating that participant engagement in this real-world citizen science project was robust to practical challenges.

**Conclusions:**

Our findings highlight the potential of diverse bee communities to mitigate negative effects of urbanization on crop productivity in urban community gardens and demonstrate the value of citizen science for pollination research. Future citizen science approaches should balance scientific accuracy with participant autonomy, for instance, by coordinating crop selection or fostering a systematic recording of management practices. Key lessons learned include involving target groups early in project design and ensuring clear communication of data quality standards.

**Supplementary Information:**

The online version contains supplementary material available at 10.1186/s12862-026-02507-x.

## Background

Insect pollinators including wild bees deliver important benefits to human society through the pollination of wild and cultivated plants, benefits collectively known as ecosystem services [34]. However, many pollinators are in decline globally due to factors such as urbanization and habitat loss [24, 55, 66, 73], and climate change related factors [64, 81]. As the production success of 75% of common food crops is supported by animals [40], understanding pollinator diversity and their associated ecosystem services is crucial for both food production and biodiversity preservation. Urban gardens are valuable study systems for pollination research because they intersect biodiversity [4, 17, 56, 74, 78] and food production [52, 54]. In these systems, gardeners’ decisions and actions can significantly influence resources for pollinators [74] and the provision of pollination services [31].

Empirical studies on biodiversity-mediated pollination in urban gardens have linked higher pollinator species richness to increased fruit set (i.e., the proportion of flowers developing into fruits) or increased seed numbers of common garden crops. For example, increased fruit set was shown for cucumber (*Cucumis sativus*), eggplant (*Solanum melongena*), and purple coneflower (*Echinacea purpurea*) [46], and increased seed numbers were shown in jalapeño (*Capsicum annuum*) [15] and pak choi (*Brassica rapa*) [53]. While the positive influence of pollinator diversity on these indicators of pollination (i.e., plant reproductive success) is relatively well documented, the influence of urbanization on this relationship is not yet fully understood. Although environmental variables associated with urbanization, such as higher temperatures or changes in land cover, can affect pollinator biodiversity, a recent review found that the majority of articles examined did not find any negative effects of urbanization on the pollination of native plants [83]. In some cases, pollination services can even be enhanced in cities compared to their rural surroundings as pollinator effectiveness (i.e., more seeds per flower of grassland legumes) can be higher [75] and pollination activity can be prolonged in spring and autumn [86]. However, several studies also report negative effects of urbanization on pollinators and pollination. Increased landscape imperviousness can reduce nesting opportunities for bees [6] and decrease habitat connectivity [3], leading to lower flower visitation rates. Elevated temperatures in densely built areas may further disrupt the temporal synchrony between flowering plants and their pollinators, resulting in phenological mismatches [26]. Urbanization can also directly affect plants and their reproductive success: higher temperatures and drought stress have been associated with reduced fruit development and lower crop yields in urban gardens [53], as well as decreased growth and flower production in grassland legumes [75].

Public interest in pollinator conservation is generally high [35]. Yet, public understanding of pollinator diversity and ecological roles remains limited [58]. Citizen science holds potential for education and scientific engagement on pollinator ecology and conservation [13], especially in systems like gardens, where relationships between biodiversity and ecosystem services should be observed under real-world conditions [1]. Following Bonney et al., [9], citizen science can be defined as a collaborative approach to research that actively involves members of the public in creating scientific knowledge, enhancing participants’ self-reported knowledge and self-efficacy (also supported by [61]) and benefiting nature connectedness [62].

Citizen science has been increasingly used to monitor pollinator populations because it can engage large numbers of volunteers and generate long-term data (UK Pollinator Monitoring Scheme [80, 11]). While this approach provides valuable insights into pollinator populations, it also faces several challenges. Citizen science data can show temporal bias due to uneven sampling effort across time, and spatial bias, as observations concentrate in accessible or populated areas (see e.g., [27]). Moreover, taxonomic identification remains difficult for certain groups, and expert verification capacity is limited [27]. Other citizen science approaches have gone beyond observational surveys by involving participants in experimental designs applied to investigate pollinator diversity. For example, Griffiths-Lee et al., [32] engaged volunteers in planting wildflowers in their private gardens or allotments and setting up traps and collecting insect samples. Their findings provided evidence that small-scale floral enhancements can attract beneficial insects. After the first year of the two-year study period, however, only 45% of participants returned samples. The authors suggested that the loss of participants was not random, but that participants with poorly established wildflowers or low insect catches were more likely to leave the project, while those with more pollinator-friendly gardens remained engaged. Their finding shows that perceived success of an experimental approach can influence the participants motivation to further contribute, which possibly introduces a bias to the data.

Several studies have demonstrated the potential of citizen science to contribute valuable data on pollination services. For example, Birkin and Goulson [7] showed that volunteers can successfully apply standardized pollination treatments to broad beans (*Vicia faba*) to measure pollination. In contrast, a hybrid study in Seattle community gardens that combined manipulative experiments with citizen participation found that pollinator visitation rates and tomato (*Solanum lycopersicum*) fruit size declined with increasing landscape imperviousness [82]. Similarly, Garratt et al., [29, 31, 41] confirmed that citizen scientists are capable of implementing structured pollination experiments in both agricultural and garden settings. However, these studies also highlight the practical challenges of such approaches. For instance, Clifford and Waters, [14] reported that low participation rates limited the robustness of their citizen-generated data, requiring additional data collection by trained interns. Across studies, difficulties related to plant health, crop loss or complex protocols have been noted as factors that can hinder data collection or continuity of participation (e.g., [31, 41]. Participation itself is not a fixed attribute but a dynamic process that varies in duration and intensity with multiple entry and exit points throughout a project [25]. The framework by Fischer et al., [25] distinguishes between natural and premature endings of participation, emphasizing that perceived challenges encountered during the process can lead to participant drop-out. Despite the proven potential of citizen science to generate meaningful pollination data, understanding what affects participation is therefore important to improve both data return and participant retention in citizen science.

In our study, we examine the relationship between biodiversity and ecosystem service provision by using citizen science as a research method to investigate how bee diversity influences the fruit set of crops commonly grown in urban community gardens. By involving gardeners directly in the research process, we seek to complement controlled experimental studies with insights obtained under real-world gardening conditions. To better understand participation dynamics, we also examine which participation types, defined by the duration and frequency of data collection, occurred in our project, how these types impacted the collected data and whether they were influenced by challenges such as plant health, harvest loss or the data collection protocol. Through the integration of ecological and citizen science perspectives, we aim to advance both the conceptual development and practical application of citizen science in real-world pollination research.

Specifically, we asked:

1. What is the relationship between bee diversity, urbanization and the fruit set of common garden crops in urban community gardens?
  - What is the relationship between bee species richness and fruit set?
  - How do urbanization factors, i.e., landscape imperviousness and temperature, affect fruit set?

We predicted that the plants studied in gardens with greater bee diversity (i.e., higher number of bee species) would have a higher fruit set (i.e., the proportion of flowers developing into fruits) than plants in gardens with lower bee species richness due to higher pollination rates. Specifically, we expected plant taxa that are highly dependent on insect pollination, such as *Cucurbitaceae*, to exhibit higher fruit set when bee diversity was higher. Conversely, we expected that plants in more urbanized gardens would have lower fruit set due to the negative effects of urban stressors on pollinators and plants. As other studies have shown (e.g., [75]), we hypothesized that the negative effects of urbanization outweigh the benefits of a higher number of bee species.

2) What types of participation, defined by the duration and frequency of data collection, occur in the citizen science project and how are they related to the collected data as well as to participant-reported challenges?
  - What is the impact of the participation types on the collected data, i.e., fruit set?
  - How are the participation types influenced by challenges such as plant health, harvest loss and the citizen science data collection protocol?

Following Fischer et al., [25], we predicted that the low entry barriers of our approach would attract high levels of participants who contribute only briefly (short and/or low frequency) and fewer who remain consistently active. However, we expected our method to accommodate varying types of participation resulting in comparable fruit set values. Based on the findings of Griffiths-Lee et al., [31], who reported that over half of participants discontinued after their plants died, we expected similar challenges to affect participation in our project. Poor plant health, harvest loss, or difficulties with the citizen science protocol can reduce motivation or make data collection impossible, leading to less frequent or shorter participation compared with gardeners whose plants remained healthy and whose data collection proceeded smoothly.

## Methods

### Study sites

The study was conducted over two years (2020 and 2021) in 22 community gardens in Berlin (52.5200° N, 13.4050° E, 891.12 km²) and Munich (48.1351° N, 11.5820° E, 310.70 km²), Germany. Berlin has a population of approximately 3.8 million [76] and the mean annual temperature and precipitation are 10.7 °C and 580.1 mm, respectively (1991–2020; [18, 19]). Munich has approximately 1.5 million inhabitants [76] and a mean annual temperature of 10.1 °C with a precipitation of 939.7 mm (1991–2020; Deutscher Wetterdienst [18, 19]). In Berlin, we studied 14 gardens (eleven in 2020 and eight in 2021, with five gardens replaced by two others in 2021) and in Munich, we studied eight gardens (all in 2021). The gardens were distributed over the two cities to acquire a wide urbanization gradient (ranging from 20.9 to 96.2% landscape imperviousness). The size of a garden was defined by the allotment boundaries and measured using Open Street Maps Tiles and satellite images in ArcGIS 10.5.1 [22], QGIS 3.32 [67] and Google Maps. Gardens varied in size from 0.04 to 2.39 ha (Supplementary Information: Table S1).

### Citizen science data collection of fruit set

Gardeners were invited to participate in the data collection via displaying a poster in the gardens and emailing individual gardeners who acted as our contact persons for the gardens. Exchange and contact between the gardeners and scientists took place mainly online due to the COVID-19 pandemic in three organized project introduction and Q&A sessions and during our garden visits for recording bees in the gardens. We also provided the gardeners with written and illustrated instructions and prepared data sheets. Participation was voluntary and limited to adults (≥ 18 years). Participants could choose whether to provide their name or remain anonymous; personal information was optional and not used in the analysis. All data were analyzed in anonymized form and handled in accordance with the General Data Protection Regulation (GDPR). A formal ethics approval was not required for this type of non-interventional, anonymized research.

We asked community gardeners to document the development of one or more plants from a list of the common garden crops: cucumber, zucchini, pumpkin, strawberry, tomato, pepper, chili and pepperoni. We chose to study these eight garden crops because we expected them to be frequently grown in these studied urban community gardens [72]. In addition, we wanted to give gardeners freedom in their choice of crop. We also expected these crops to benefit from insect pollination as shown for cucumber (*Cucumis sativus*) [46], pumpkin (*Cucurbita moschata*) [37], tomato (*Solanum lycopersicon*) [2], strawberry (*Fragaria x ananassa*) [57] and pepper plants (*Capsicum annuum*) [15]. *Cucurbitaceae* (cucumber, pumpkin and zucchini) are monoecious (both male and female flowers on the same plant) and rely strongly on insect pollination, while strawberry, tomato, pepper, pepperoni and chili are capable of self-pollinating but nevertheless benefit from insect pollination. In garden strawberry (*Fragaria x ananassa*), for example, insect pollination led to improved yield (i.e., bigger fruits) and higher marketable quality of fruits (e.g., fruit appearance, variety-specific taste and shelf life) compared to self-pollination [85]. In communicating with gardeners, we did not give instructions on specific species or varieties and therefore did not use scientific species names.

In the standardized data sheet, gardeners documented the number of observed open flowers, faded flowers, fruits on the plant and fruits they harvested. To ensure consistent identification of flowering stages, the protocol provided illustrative images. The images showed an open, fresh flower with vivid color, a closed flower displaying senescence indicators such as brownish coloration, limp or fallen petals, or early fruit formation, and fruits at both unripe and ripe stages. Furthermore, they noted the date, time, name of observer, and additional comments on the data sheets. Participants were encouraged to use the comment field to report any additional observations or relevant information about the plant, study conditions or protocol. However, the field was open-ended and no specific instructions were provided, so comments were not created or recorded in a standardized way. We instructed them to maintain observations with a constant time interval of every three to four days over the course of the growing season for at least eight weeks, but did not mandate a start or end date for the measurement. Participants were advised that it is particularly important to mark the plant and to always measure in the same way to establish comparability between their measurements. To examine the variability of fruit set in a real-world gardening context, no standardized management practices (e.g., irrigation or fertilization) were imposed. Instead, we relied on gardeners’ customary practices adapted to their specific local conditions.

To calculate the fruit set, we then defined standardized data quality specifications and excluded all datasets that did not meet the following criteria:

1. The observed plant was one of the eight selected crops.
2. The observation series included at least three measurements.
3. There was no fruit at the first measurement (as indicator that no development stages were missed).
4. The observation series was plausible and without errors (e.g., fruits without ever documenting flowers, missing or wrong data or similar).
5. There were at least three study plants per garden and year (independent from crop type).

Additionally, we also excluded single data points when we found irregularities, for example typos in the number of harvested fruits. We did not exclude observation series where no fruit developed, if they met all other quality criteria. With the remaining data we calculated the fruit set based on Bennett and Lovell [6] as the number of fruits divided by the number of flowers that bloomed on an individual plant multiplied by 100.

$$Fruit\:set\left(\%\right)=\left(\frac{Number\:of\:fruits}{Number\:of\:flowers}\right)\cdot100$$

For this, we calculated the number of fruits by calculating the difference between the number of fruits at the time of measurement and the number of fruits at the previous time of measurement minus the harvested fruits that were removed from the plant. All differences were added to obtain the total number of fruits. For some plant observations, fruits were harvested before counting: the number of harvested fruits was higher than the number of fruits on the plant, or a gardener explicitly mentioned this method in the comments. If gardeners used this method of harvesting first and then counting the fruits for one plant observation, we assumed they remained consistent with one procedure for all their observations. For these observation series we calculated the total number of fruits by calculating the number at the time of measurement (on the plant plus harvested) minus the number of fruits remaining on the plant since the previous time of measurement and summed up all differences. In both cases negative differences indicated the loss of fruit during the measurement period and were therefore treated as no additional fruit. With this approach, we were able to ensure that fruits that stayed on the plant for several measurement times were not included in the total more than once.

We also calculated the number of flowers by calculating the difference between open and faded flowers at the time of the measurement and at the previous measurement time to ensure that flowers remaining on the plant were not counted multiple times. To consider the further development of the flowers, we subtracted all faded flowers that have developed into fruits in the meantime and all open flowers that have faded or also developed into fruits from the number of the respective flower stage at the previous measurement time. All differences were added to the total sum of flowers. To reduce a possible bias due to unmeasured flowers because of missed stages, we also added to the total sum all fruits or faded flowers whose emergence from a previous stage could not be verified. It should be noted that besides our best efforts to account for missed stages, it is possible that flowers opened, faded and fell off the plant unnoticed between two measurements. Open and faded flowers that already existed at the beginning of a measurement series were also added. As the successful pollination of newly opened and faded flowers at the last measurement cannot be examined, these flowers were subtracted from the total. For calculating the number of flowers, we did not ask the gardeners to differentiate between male and female flowers in *Cucurbitaceae* in order to keep the protocol as simple as possible.

### Bee observations

To assess bee diversity across all 22 community gardens, we visited each garden two to four times per participation year between April and August. We established a 20 × 20 m plot in the center of each garden and adjusted the plot to 10 × 40 m, when the garden shape did not allow for a 20 × 20 m plot (i.e., gardens were more narrow than wide). In 2020, the plots were divided into four parallel transects of 5 × 20 m each, along which two observers walked the transects slowly, pausing regularly to survey flowers for bees. All observations were performed by two observers to avoid observer bias and between 8:00 and 18:00 during good weather conditions (no rain and temperatures above 10 °C). In 2020, all bees on flowers and in flight were counted and identified as accurately as possible at the order, family, or genus level for a total observation time of 60 min. Individuals that could not be identified to the species level in the field were netted, transferred to plastic containers previously covered with formalin-soaked absorbent cotton, and taken to the laboratory for species identification. The observation time was paused during handling of individuals to maintain the 60 min observation and to avoid double counting individuals [23]. In 2021, as explained in Neumann et al., [56], we recorded all bees on flowers, on the ground and in flight for 30 min along transects covering the whole 20 × 20 m plot. Individual bees that could not be identified to the species level in the field were collected using ethyl acetate and taken to the laboratory for species identification. Observation time was paused during handling time for capturing.

For further analysis, we calculated bee richness as the sum of all bee species that were identified to species level, including the managed honeybee (*Apis mellifera*), per garden and year and bee abundance as the sum of all bee individuals that were identified to species level, also including the managed honeybee (*Apis mellifera*), per garden and year.

### Urbanization factors

Following Neumann et al., [56], we measured urbanization around the gardens as the percentage of impervious surfaces using ArcGIS 10.5.1 (ESRI 2017). We calculated the landscape imperviousness within a buffer of a 1000 m radius, centered in the 400 m² plot. As datasets we used Copernicus High Resolution Layer: Imperviousness Density (IMD) 2018 (European Union, Copernicus Land Monitoring Service 2020, European Environment Agency (EEA) with a resolution of 10 × 10 m. Since increased temperature is often associated with urbanization [60] and possibly affects pollinators [86] and plant development [75], we also measured the average temperature within each garden with an Onset HOBO data logger (Onset HOBO UA-002064). Three weeks prior to the first sampling period, temperature loggers were placed two meters above ground on a pole in the center of the garden and set to record hourly averaged ambient temperature. We collected the data at the end of the study using an Optic USB interface, and quality-checked and cleaned the data [57]. The average temperature per garden and per year was calculated from daily average temperatures for each day during the growing season from June to September.

### Participation types

Based on the framework by Fischer et al., [25], we used the duration and frequency of participation to group all citizen science contributions (i.e., observed plants) into participation types. We focused our analysis on individual plants rather than participants, as we were interested in plant-specific challenges and their effect on participation. We calculated the duration as the total number of days from the first to the last measurement for each examined plant. For participation frequency the number of days between the first and last measurement was divided by the total number of measurements. According to the protocol, participants were asked to collect data for at least eight weeks and every three to four days. Consequently, a total of 56 days was used as the threshold to distinguish between short and long participation duration, and an average interval of every four days was used to differentiate between low and high measurement frequency. For further analyses, contributions were classified into four participation types based on the combination of duration and frequency of measurements: (1) short duration and low frequency, (2) short duration and high frequency, (3) long duration and low frequency, and (4) long duration and high frequency. Contributions consisting of a single observation were excluded from these types.

### Participants’ comments

Based on previous literature [31, 41], we identified three main challenges related to participation: plant health issues, harvest loss, and difficulties with the data collection protocol. To explore how these challenges were reflected in participants’ comments, we conducted a deductive qualitative content analysis following Mayring [51]. All of the open-ended comments provided by the citizen scientists were screened for statements related to these three challenges and subsequently categorized accordingly (Table 1). Additionally, we coded “Plant death” as a category because the death of a plant led to an end of participation that was not chosen by the participant. The initial coding was carried out by one researcher and independently cross-checked by a second researcher to ensure consistency and reliability. Some comments displayed additional reflective depth and were selected as illustrative examples to exemplify how participants engaged with and interpreted their own observations and experiences.

**Table 1:** Categories of challenges used for coding of comments with a brief description and a representative quote. All comments not relating to one of the three challenges or plant death were coded under “Other topics”

Category	Description	Representative quote
Plant health	Comments on negative plant health due to e.g. withering, pests or diseases of the plant.	*“Probably mice are eating the buds. The plant overall appears weakened.”*
Plant death	Comments that reported a plant to be dead.	*“Plant is dead.”*
Harvest loss	Comments on loss of fruits due to diseases or pests influencing fruits, animals eating fruits or humans stealing fruits.	*“1 rotten fruit removed”*
Citizen science protocol	Comments on an uncertainty about the methods and their own data collection or on difficulties to follow the protocol.	*“The last measurement must be 22 buds (not 9).”* *“No visit on 17 June due to extreme heat.”*
Other topics	Comments on e.g., the weather, visiting animals, plants traits or management activities including irrigation, mulching and fertilization.	*“recently tied up”* *“cold and rainy weather”* *“2 m distance from honey bees”*

Subsequently, each observation series (i.e., each monitored plant) was examined to determine whether a comment had been provided or not and which categories applied. A single observation series could include comments to one or several categories (e.g., references to harvest loss, plant health, plant death or the citizen science protocol) or none at all.

### Data analysis

We first evaluated the potential relationship between bee diversity, urbanization factors and the fruit set of the studied garden crops to address research question 1. Subsequently, to answer research question 2, we assessed the potential impact of the different types of participation on the collected data and examined how challenges reported by the citizen scientists influenced these participation types.

To evaluate the impact of bee diversity and the urbanization factors on fruit set of the selected garden crops, we fitted linear mixed-effects models (LMMs) using the lmer function from the *lmerTest* R package [5, 44], with fruit set as response variable in all models. We only included data that met our quality criteria as described above and combined the data from both study years (2020 and 2021) to increase statistical power. As fixed effects, we included bee species richness, bee abundance, degree of impervious surfaces within a 1000 m radius around the garden (landscape imperviousness) and mean air temperature during flowering season (temperature). Garden size and year, which could potentially influence pollinator diversity, were included as covariates in all models. We scaled bee species richness, bee abundance, landscape imperviousness, temperature and garden size in all models, to account for differences in sampling methods between years and measurement scales among predictors. We included garden identity (i.e. garden name) as a random effect to account for site-specific conditions such as microclimate and garden management practices. Informal conversations with participants indicated that, for example, some gardens have shared regulations on fertilization that all members are required to follow.

Because bee species richness and abundance were moderately correlated (Spearman’s *ρ* = 0.52, *p* < 0.001) and represent partly overlapping but conceptually distinct ecological properties, they were not included simultaneously in the same models. Instead, richness-based and abundance-based models were fitted separately. We primarily focused on pollinator diversity effects on fruit set using bee species richness as alpha-diversity metric, to better understand the underlying biodiversity-ecosystem service relationship [38]. Therefore, richness-based models formed the main analysis, while abundance-based models were used as complementary analyses and are reported in the Supplementary Information to ensure transparency and to test the robustness of the main results (for model structure and results see Supplementary Information: Tables S3-S6).

Moreover, to test individual relationships between bee diversity and urbanization metrics while avoiding overparameterization, interaction terms were added individually to the model, leading to the following model structure:

I. Fruit set ~ bee richness * landscape imperviousness + temperature + garden size + year + (1|garden)
II. Fruit set ~ bee richness * temperature + landscape imperviousness + garden size + year + (1|garden)

As *Cucurbitaceae* are monoecious and strongly dependent on insect pollination while the other crops are capable of self-pollinating, we divided the plants into two groups called *Cucurbitaceae* (cucumber, pumpkin and zucchini) and *Solanaceae* (tomato, pepper, pepperoni and chili plus strawberry). In the first step, all observations were analyzed jointly, adding plant group (*Solanaceae* vs. *Cucurbitaceae*) as a fixed factor. Because *Solanaceae* and *Cucurbitaceae* differ fundamentally in reproductive biology and we hypothesized that this would lead to differences in the effects of predictor variables on the crop-specific response variables, we subsequently conducted separate analyses for each crop group. This allowed us to test the hypothesis that the relationship between bee diversity and fruit set differ between plant families.

Model assumptions were assessed by visually inspecting residual plots, including Q-Q-plots and plots of residuals versus fitted values, and by testing for normality of residuals using the Shapiro-Wilk test. Moreover, standardized residuals were calculated to assess the distribution of model residuals and values lay within the commonly accepted range of ± 3. Multicollinearity was examined using variance inflation factors (VIF) [28], and all values were below 5.

To assess whether the four participation types impacted the data collected (i.e., fruit set), a one-way analysis of variance (ANOVA) was performed. The participation types were treated as a categorical independent variable with fruit set as response variable. The assumptions of ANOVA were checked using Q-Q plots and Levene’s test [28] and were met. For any significant results, post-hoc pairwise comparisons were conducted using Tukey’s Honestly Significant Difference (TukeyHSD) test to identify specific differences between groups. Again, we first analyzed all data together and subsequently repeated the analysis for the two plant groups (*Solanaceae* and *Cucurbitaceae*) separately. We included only contributions that were in the final data set for the fruit set analysis as other contributions that did not meet our quality criteria were already excluded because of their potential impact on the data.

To examine if challenges reported in the participants’ comments influenced different types of participation, we conducted Fisher’s exact tests for identifying differences between participation types for each challenge category, i.e., harvest loss, difficulties with plant health, and protocol-related issues. We included all contributions, regardless of whether they met our quality criteria for the fruit set analysis or not and followed the same analysis pattern as before (first a joint analysis of all data and then separately by plant group). For any significant results, post-hoc pairwise comparisons with Holm correction would have been performed using the pairwiseNominalIndependence function from the *rcompanion* R package [49]. Since none of the Fisher tests were significant, no post-hoc analyses were conducted.

All analyses were performed in the R Statistical Environment (version 4.4.2) [68] and RStudio (version 2024.12.1.563) [63]. Results were visualized using the *ggplot2* R package [84].

## Results

### Citizen science data on fruit set

**Fig. 1:**
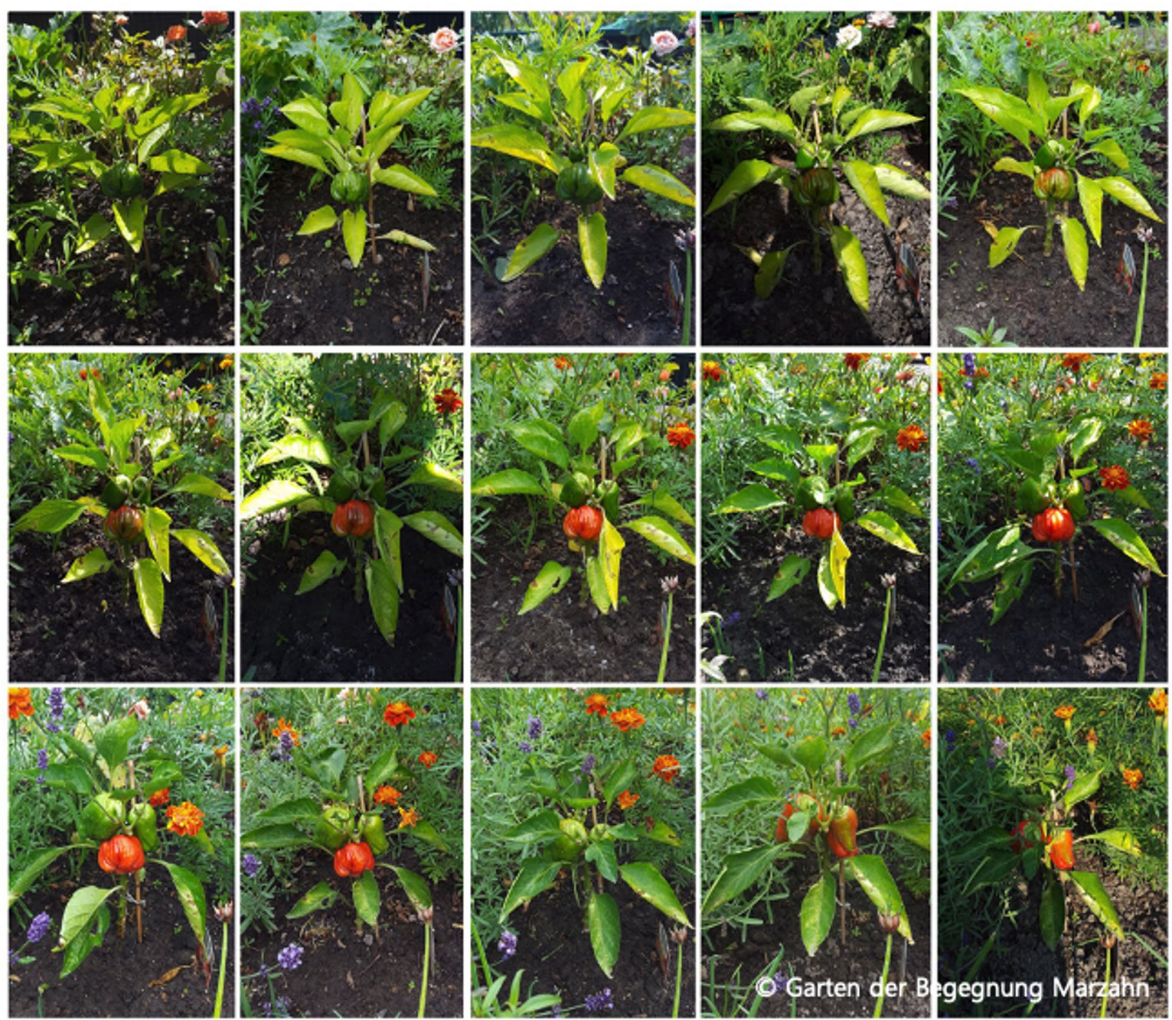
Example of a studied pepper plant. Some gardeners sent us pictures of their plants’ development throughout the season for an accompanying photo project (photos courtesy of a gardener (name withheld) from Garten der Begegnung Marzahn)

In total, 73 citizen scientists from 22 community gardens in Berlin and Munich examined 150 plants (Fig. 1). From informal conversations with gardeners, we estimate that this is about a 20% participation rate across all 22 gardens (a total of approx. 760 gardeners are gardening in these gardens). Overall, each crop type from the selection and in addition one physalis plant were examined. Most citizen scientists decided to study tomato plants (34% of all observed plants). Of the 150 study plants, 61 did not produce any harvestable yield and 17 of them did not produce any fruit at all [39].

We first performed a rigid cleaning of the data according to our quality criteria, which led to the exclusion of 57 observed plants (38% of the total, Table 2). After the data cleaning process, our final data set included all of the eight crops except chili with observations from 14 community gardens. Ten of these gardens were located in Berlin and four gardens in Munich. Furthermore, the final data set included 93 observed plants, 47 in the group *Cucurbitaceae* and 46 in the group *Solanaceae* (including strawberries). Fruit set varied from 0 to 87.5% (mean = 35.7%, ± 21.0 SD) in *Cucurbitaceae* and from 0 to 93.9% (mean = 58.4%, ± 22.5 SD) in *Solanaceae* (Supplementary Information: Table S2).

**Table 2:** Quality criteria applied to the data set on fruit set in urban community gardens

Quality criterion	Number of contributions that did not meet the criterion
The observed plant was one of the eight selected crops	2
The observation series included at least three measurements	4
No fruit at the first measurement	22
The observation series was plausible and without errors	10
At least three study plants per garden and year	19
**Total**	**57**

### Pollinator diversity, urbanization factors and their impacts on fruit set

Over the two study years in the 14 gardens left in the final data set, we observed an average bee species richness of 25.2 (± 7.5 SD) and bee abundance of 153.7 (± 122.8 SD) per garden. Landscape imperviousness in a 1000 m radius of these 14 gardens varied from 23.9 to 96.2% and the average temperature per garden ranged from 18.4 to 21.5 °C (Supplementary Information: Table S1).

Across the full dataset, two linear mixed-effects models were fitted, each including a single interaction term between bee species richness and landscape imperviousness or temperature (Supplementary Information: Table S3). Plant family was a significant predictor in both models, with higher fruit set in *Solanaceae* compared to *Cucurbitaceae* (*p* < 0.001). The interaction between bee richness and temperature was significant (*p* = 0.026). Conditional R² values ranged from 0.41 to 0.49 (Supplementary Information: Table S6).

**Fig. 2:**
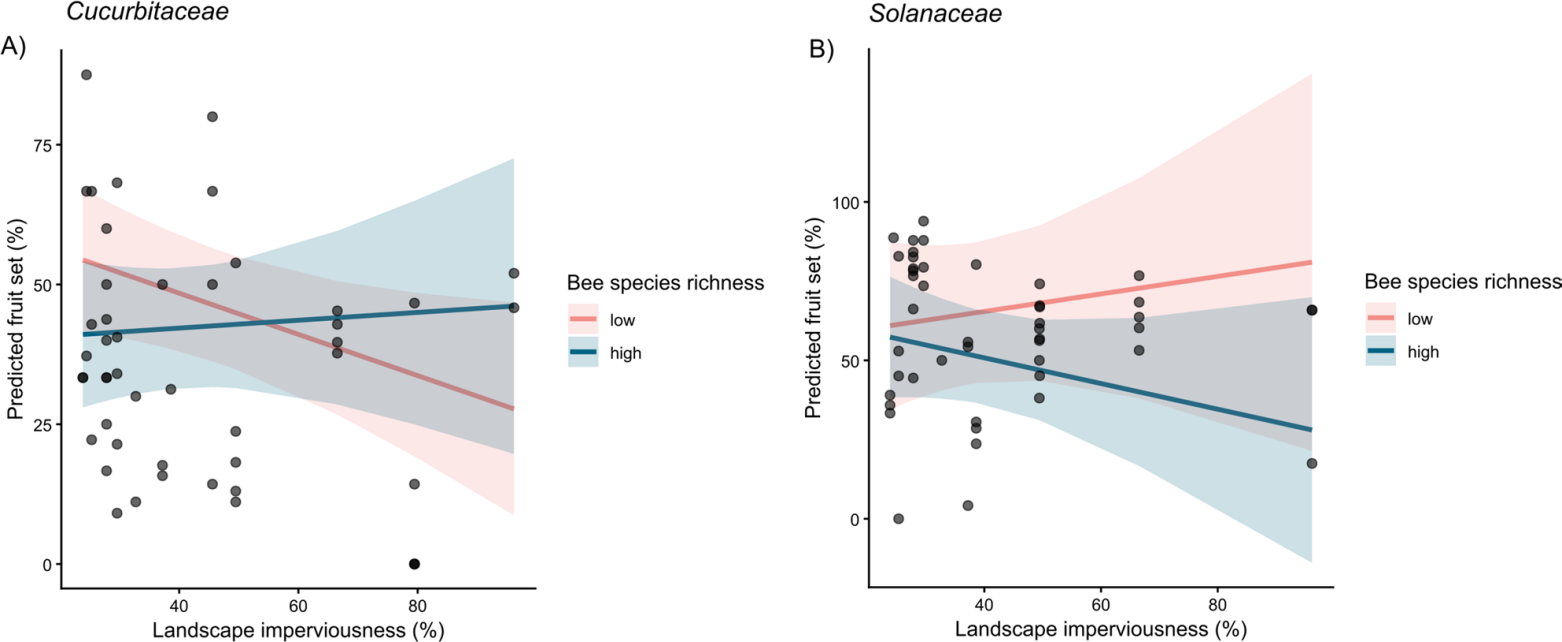
Model-predicted effects of the interaction between bee species richness and landscape imperviousness for **A**) *Cucurbitaceae* and **B**) *Solanaceae* based on linear mixed-effects models. Predictions are shown across the observed range of imperviousness for two representative richness levels (25th and 75th percentile), while all other predictors were held constant. Solid lines indicate fitted relationships and shaded areas represent 95% confidence intervals. Black points represent raw observed fruit set data. Model predictions were generated using the ggpredict function from the *ggeffects* R package [47]

For *Cucurbitaceae*, models were fitted including the same interaction structure as in the full dataset (Supplementary Information: Table S4). While no predictor alone showed a significant main effect, a significant interaction was found between bee species richness and landscape imperviousness (*p* = 0.040) (Fig. 2A). Conditional R² values ranged from 0.22 to 0.25 (Supplementary Information: Table S6). In contrast, for *Solanaceae*, none of the main effects or interaction terms were statistically significant (Fig. 2B, Supplementary Information: Table S5) with conditional R² was slightly above 0.40 in both models (Supplementary Information: Table S6).

Complementary analyses using bee abundance instead of species richness produced broadly comparable qualitative patterns across datasets. While abundance-based models occasionally showed slightly improved model fit (lower AIC values), the overall structure of effects and ecological interpretation remained consistent. Abundance-based model results are presented in detail in the Supplementary Information (Supplementary Information: Tables S3-S6).

### Participation types, their impact on the collected data and the influence of participant-reported challenges

**Fig. 3:**
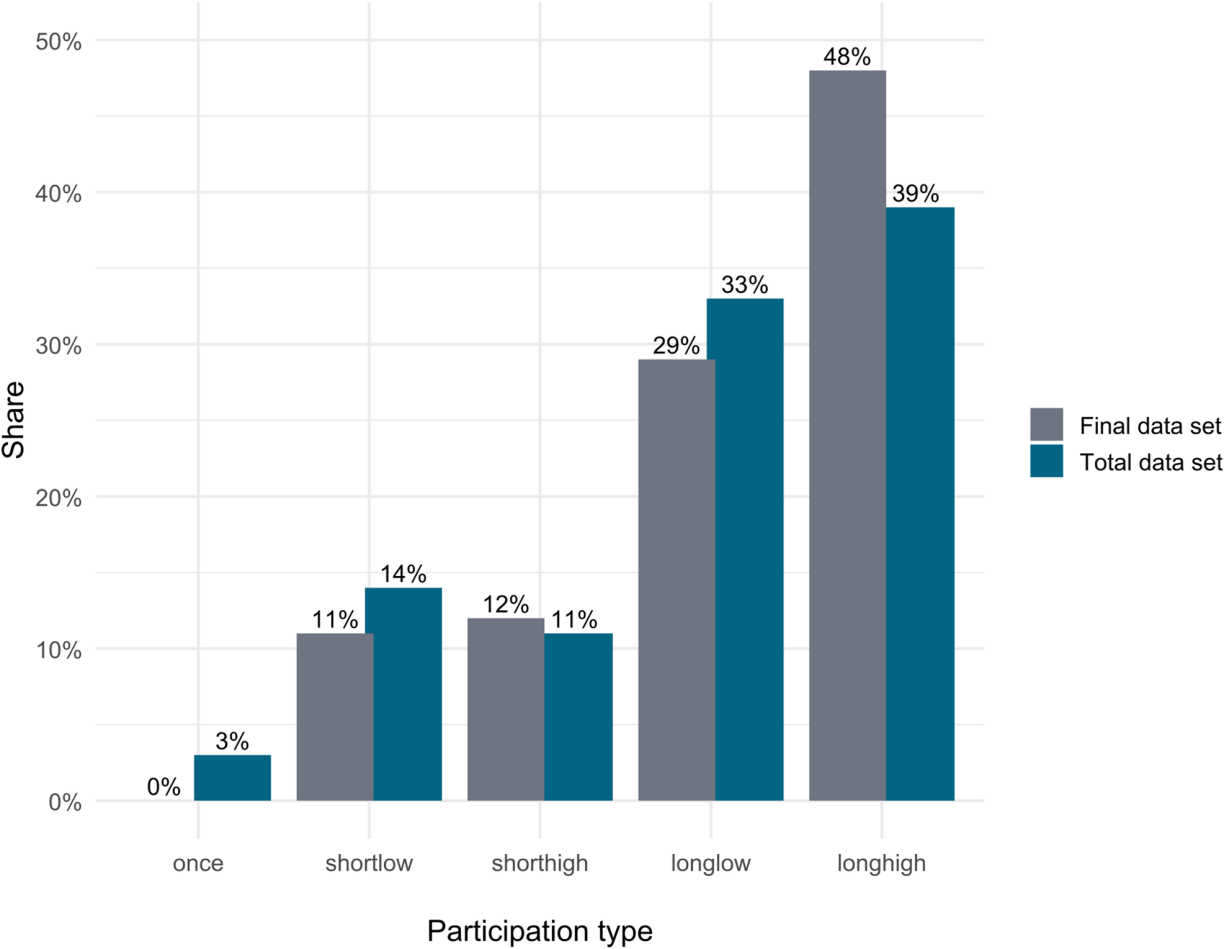
Share of the different participation types in the total data set (*n* = 150 observed plants) and in the final data set used for analysis (*n* = 93). Abbreviations: once = single observation, shortlow = short duration, low frequency, shorthigh = short duration, high frequency, longlow = long duration, low frequency, longhigh = long duration, high frequency

Of the 150 observation series submitted, five included only one measurement. Approximately 25% (*n* = 37) of the series were shorter and over 70% (*n* = 108) were longer than the eight weeks proposed in the protocol (Table 3; Fig. 3). About half of all plants were measured at the intended frequency of every four days or less (*n* = 74).

**Table 3:** Participation types, total number of contributions, mean duration (in days from the first to the last measurement ± standard deviation) and mean frequency (in days between consecutive measurements ± standard deviation) of participation per type. Participation types were defined based on the combination of duration and frequency of participation. Contributions consisting of a single observation were not included

Participation type	Number of contributions	Duration (± SD)	Frequency (± SD)
Short duration, low frequency	21	41 (± 12.37)	5.84 (± 0.77)
Short duration, high frequency	16	35.88 (± 12.29)	3.54 (± 0.45)
Long duration, low frequency	50	81.28 (± 18.75)	5.99 (± 1.14)
Long duration, high frequency	58	92.12 (± 11.49)	3.69 (± 0.55)

After data cleaning, 93 observation series remained, representing all four participation types: 45 long duration and high frequency, 27 long duration and low frequency, 11 short duration and high frequency, and 10 short duration and low frequency (Fig. 3). Overall, participants took a break of more than ten days between measurements in 43 of the 93 observation series used in the analysis. Most of these long breaks occurred in the long-duration, low-frequency participation type (*n* = 18), representing 67% of the plants in this group and 42% of all observation series with such breaks.

When analyzing the impact of the participation types on the fruit set data of all contributions combined, the mean fruit set ranged from 40.2 to 53.6% (Table 4). For *Cucurbitaceae*, means ranged from 30.5 to 38.6%. For the group *Solanaceae*, the mean fruit set was highest when participants measured over a long time period and at least every four days (70.6%) and lowest when participants measured over a short time and less frequent than every four days (45.4%).

**Table 4:** Mean fruit set ± standard deviation of all contributions (i.e., study plants examined) combined, study plants in the group *Cucurbitaceae* and study plants in the group *Solanaceae* per participation type

Participation type	Fruit set all contributions (± SD)	Fruit set *Cucurbitaceae* (± SD)	Fruit set *Solanaceae* (± SD)
Short duration, low frequency	40.2 (± 31.7)	35.0 (± 35.7)	45.4 (± 30.2)
Short duration, high frequency	41.4 (± 21.7)	35.4 (± 17.3)	46.5 (± 25.1)
Long duration, low frequency	40.5 (± 23.1)	30.5 (± 20.5)	49.8 (± 22.2)
Long duration, high frequency	53.6 (± 23.1)	38.6 (± 19.0)	70.6 (± 13.6)

**Fig. 4:**
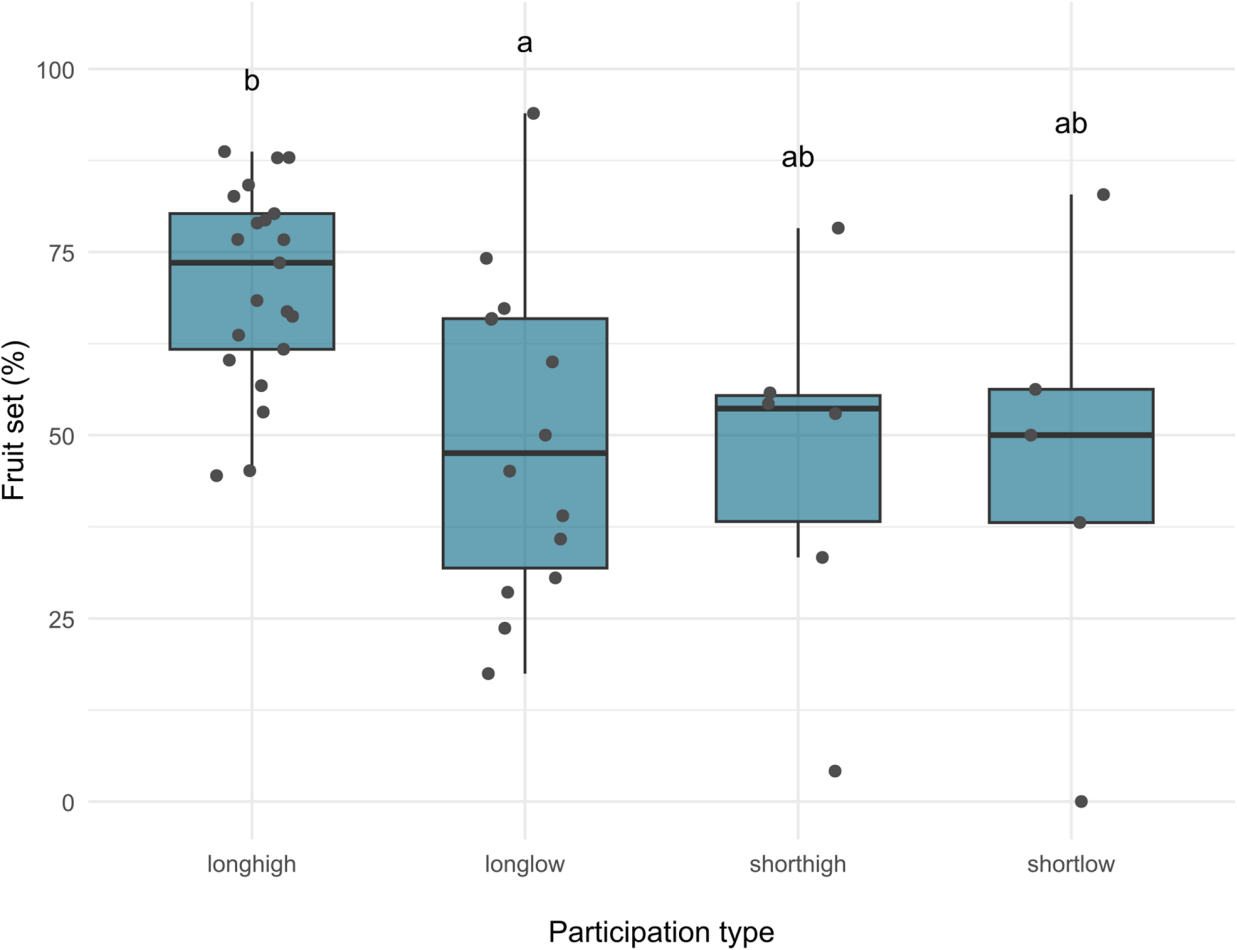
Fruit set across participation types. Boxplots show median, interquartile range, and data distribution. Points represent individual observations. Groups sharing the same letter are not significantly different (Tukey HSD, *p* < 0.05). Letters were generated using the multcompLetters function from the *multcompView* R package [30]. Abbreviations: shortlow = short duration, low frequency, shorthigh = short duration, high frequency, longlow = long duration, low frequency, longhigh = long duration, high frequency

The one-way ANOVA revealed a significant difference of fruit set between participation types only for the group *Solanaceae* (*p* = 0.005), whereas no significant differences were found for either all contributions combined or the group *Cucurbitaceae*. Post-hoc Tukey tests for *Solanaceae* indicated that the participant type with a long duration and high frequency of measurements differed significantly from the long duration and low frequency type (Fig. 4), while the short duration types were not significantly different from either of the long duration groups.

Comments were provided for 115 of the 150 observed plants. Challenges with plant health were reported for 35% (*n* = 52), death of the study plant for 7% (*n* = 11), harvest loss for about 30% (*n* = 46) and protocol-related issues for 25% (*n* = 37) of observations. Of the 46 crops for which harvest losses were reported, participants cited fruit theft in 11 cases. For 28 study plants, participants’ comments contained information on topics other than the three challenges or plant mortality. All participation types included comments on each of the three challenge categories. Participants with low-frequency contributions most frequently commented on plant health, while those with long duration and high frequency most often mentioned difficulties with the protocol. Contributions of short duration and high frequency contained a balanced distribution of comments on issues with plant health and harvest loss (Table 5, for results for *Cucurbitacea* and *Solanaceae* including strawberries see Supplementary Information: Table S7 and S8). Fisher’s exact tests revealed no significant differences between participation types regarding the share of contributions with comments related to harvest loss, plant health, or protocol issues, either in the analysis of all data together or in the analysis separated by plant group.

**Table 5:** Share of contributions (i.e., study plants examined) per participation type that included a comment on plant health, plant death, harvest loss and the protocol in %. Note: A contribution may contain comments relating to several categories, which means that the total percentage of all categories may exceed 100%

Participation type	Plant health	Plant death	Harvest loss	Citizen science protocol
Short duration, low frequency	29	24	19	14
Short duration, high frequency	38	0	38	19
Long duration, low frequency	42	10	36	20
Long duration, high frequency	33	2	31	36

Several comments also contained reflective summaries, in which participants described both plant performance and contextual factors such as weather conditions or management decisions. One gardener noted as summary:

*Overall, the plant may not best represent pollination; it self-seeded*,* was later transplanted*,* and took a long time to recover. It is infected with mildew*,* and the very hot July certainly did not help. At the moment*,* I expect to harvest only two fruits - other fruits and flowers have dried out.*

Similarly, other participants provided reflective summaries of their observations, describing both plant development and the conditions affecting pollination from their perspective:

*End of measurements because the owners removed the flowers to help the remaining tomatoes ripen. This season started six weeks later. The variety Phantasia produced very large fruits that took a long time to ripen. I never saw pollinators at the flowers, but they must have come quickly, as the flowers were already wilted when I arrived. Most fruits ripened later indoors.*

as well as

*Fruits ripe for harvest*,* 14 cm and 14.5 cm*,* premature termination of observation after 5 weeks. Today*,* on 7 July*,* I visited the plant again and had* [name of a person] *confirm that nothing more is happening there (approx. 1.5 m further away*,* there is a plant that is developing magnificently*,* facing south). Note: Location may not be ideal*,* with a feathery plant growing rampantly to the south. P.S. Unfortunately*,* I did not observe any wild bees visiting the plant (but there are plenty in the lime trees*,* where you can hear them buzzing!).*

In summary, participants frequently noted circumstances affecting fruit development, including for example delayed flowering, plant relocation, or interventions by other gardeners. Some also highlighted potential limitations in pollinator observations or environmental constraints at their site. Overall, these reflective comments provide qualitative insights into how participants perceived plant performance and the factors influencing their measurements.

## Discussion

The aim of this study was to explore how bee diversity and urbanization factors, i.e., landscape imperviousness and temperature, influence the fruit set of common garden crops under real-world gardening conditions in urban community gardens. Moreover, we aimed to better understand how participation types affect the data collected and if participation was influenced by challenges such as maintaining plant health or the data collection protocol.

Out of 150 citizen science observation series on different garden crops, 93 observations from all participation types met our quality criteria and were used for the analysis of fruit set. We found a significant positive interaction between bee species richness and the amount of impervious surface cover within a 1000 m buffer around the gardens for the fruit set of *Cucurbitaceae* crops. This follows other work in urban gardens emphasizing the importance of local wild bee communities for pollination service provisioning [46]. In contrast, no significant ecological predictors of fruit set were detected for crops in the group *Solanaceae*. However, the measured fruit set differed significantly between long duration contributions with high versus low measurement frequency. This finding may be indicative of an observer bias for this group, a frequently documented problem in citizen science data (e.g., [20]). Overall, our results suggest that different types of participation in citizen science projects have the potential to contribute useful data to pollination research, but that methodological limitations of varying intensity may exist for different crop groups.

Here we discuss our key findings on pollination services and fruit production in urban community gardens, as well as on types of participation and their impact on the collected data.

### The relationship between pollinator diversity, urbanization and fruit set

Neither bee species richness nor the environmental factors relating to garden size or urbanization alone had a significant effect on the fruit set of the studied crops in urban community gardens. In addition, temporal variation among years was not significant. However, the significant interaction between bee species richness and landscape imperviousness suggests that bee species richness plays an increasingly important role for fruit production under higher levels of urbanization. The fruit set of *Cucurbitaceae* was the lowest in gardens in highly urbanized areas with fewer bee species present. This result aligns with previous work showing a negative influence of urbanization on crop yield [53] and plant fitness [75], potentially due to heat and drought stress associated with the urban heat island phenomenon. However, we found no evidence for a negative effect of increased temperatures in more densely built-up urban areas, as temperature was not a significant predictor in any of our models.

When bee species richness was high in an urban garden, fruit set was also high, suggesting that the negative effects of environmental factors like landscape imperviousness might be mitigated by enhanced pollination. The interaction may be explained by greater functional complementarity in more diverse bee communities in urban gardens, increasing the probability of including efficient pollinators for *Cucurbitaceae* [37]. Gardens in more densely urbanized areas of the city might act as islands in the less hospitable urban matrix, causing the bees to concentrate their foraging efforts on plants inside these gardens [50]. This “concentration effect” was shown in several studies that highlight the positive effects of floral dense patches on pollination in urban agroecosystems [15, 46, 65]. Although we did not include plant richness in our study, other research in our study system showed that community gardens can harbor diverse plant communities [72] and that gardeners can promote pollinators in these gardens by providing enough floral resources [56, 70].

These effects were not observed for crops in the *Solanaceae* group, where neither bee species richness, the environmental variables describing urbanization, nor their interaction significantly predicted fruit set. However, we found that the participants’ sampling frequency influenced the fruit set in *Solanaceae*, which is discussed in detail in the next subsection. Nevertheless, the weak effects of biodiversity and urbanization on fruit set in this group could also be explained by differences in pollination biology compared to crops of the *Cucurbitaceae* group. Many *Solanaceae* species, particularly tomato (*Solanum lycopersicum*) and pepper (*Capsicum annuum)*, are largely capable of self-pollinating, although insect pollination can increase yield [16, 69, 79]. In addition, key pollinators differ from those typically visiting *Cucurbitaceae* crops: cucumbers (*Cucumis sativus*) are often visited by solitary wild bees, including *Lasioglossum* species [45], whereas tomatoes are primarily pollinated by buzz-pollinating *Bombus* species [79], which are common in urban community gardens [23, 56]. Future studies could benefit from recording flower visitors directly on focal crop species in addition to assessing pollinator communities at the garden level, as implemented in previous urban pollination studies [6, 46].

Moreover, there is evidence that environmental and management factors can have crop-specific and sometimes contrasting effects on pollination outcomes. For instance, Nguyen et al., [57] showed that local temperature positively influenced fruit development in both strawberry (*Fragaria x ananassa*) and chili pepper (*Capsicum frutescens*), whereas landscape imperviousness negatively affected fruit mass only in strawberry. In contrast, high floral resource density was associated with reduced seed mass in chili pepper but had no detectable relationship with strawberry pollination. More generally, floral resource availability can produce mixed responses in pollinators and pollination services depending on functional group identity, with some taxa showing dilution effects and others concentration effects in response to resource density [38]. Such contrasting responses potentially obscure simple biodiversity-ecosystem service relationships at the community level and highlight the importance of considering crop-specific pollination ecology when evaluating pollinator-ecosystem service relationships in urban agroecosystems.

### Participation types, their impact on the collected data and the influence of participant-reported challenges

To study pollination within the complex dynamics of urban community gardens with numerous gardeners, large datasets including a wide range of participants are beneficial. Despite the potential of engaging gardeners in citizen science initiatives, participant recruitment and retention remain widespread challenges in citizen science (e.g., [8, 41]). Our findings show that while only about 20% of potential gardeners took part in the study, a majority of those who did participate stayed engaged for a long time – longer than the proposed eight weeks. This demonstrates a high level of commitment by these gardeners. Contrary to concerns raised in other studies [25], low entry barriers in our design did not primarily attract short-term contributors but instead enabled varied participation. Furthermore, participants’ comments provided qualitative insights into their experience. Many used the comment field to reflect on their plants’ development, pollination success, and gardening conditions. This indicates that the project encouraged reflections about their observations and influencing factors. Although learning outcomes were not systematically evaluated, these reflections may point to the educational potential of citizen science to stimulate curiosity and ecological awareness as suggested by Bonney et al., [10] and Schuttler et al., [71]. This outcome indicates that an open and flexible approach, adapted to the gardeners’ real conditions, can support sustained involvement even without intensive coordination, training or control.

However, our results further indicate that, contrary to our expectations and despite the long-term commitment of most participants, differences in sampling frequency led to significant differences in fruit set measurements within the *Solanaceae* group. As many observation series in the “long duration, low frequency” group included extended gaps of more than ten days between measurements, one possible explanation is that participants in this group may have missed recording fruit development before fruits were removed from the plant. Fruit theft may contribute to this bias, as it was repeatedly mentioned in participant comments. Additionally, this group included a high proportion of tomato observations. Tomatoes occur in a wide range of cultivars and fruit sizes, including small-fruited types such as cherry tomatoes, which are more likely to be removed unintentionally or deliberately during routine garden activities compared to larger fruits such as pumpkins. This may amplify under-detection and thus lead to underestimation of fruit set when observation intervals are long. Previous studies have shown that citizen science data can be sensitive to participant behavior and reporting practices. For example, participants in a plant–pollinator study overestimated insect visitation when they waited for insects to arrive or failed to submit nil observations [59].

Contrary to expectations from Birkin and Goulson [7] and Griffiths-Lee et al., [31], participant-reported challenges such as plant health, harvest loss and the citizen science protocol did not significantly affect the participation type in our study. Instead, many gardeners who faced challenges continued their observations for an extended period. Based on Sturm et al., [77], who surveyed the same group of gardeners, we know that fascination with pollinators was the most important factor motivating participation in this citizen science project. This shared motivation likely contributed to the high level of persistence observed, suggesting that emotional engagement and personal interest can buffer against practical obstacles and sustain participation over time. Unlike previous reports from other citizen science projects, where volunteers may refrain from submitting data they perceive as insufficient or incomplete [59], we did not observe evidence of such reluctance. Instead, participants frequently submitted short or partial observation series, which may reflect the inclusive design of our study. Rather than indicating a redesign of citizen science projects toward simpler tasks, our findings show the potential of adapting citizen science activities to the real-world practices and interests of participants to support long-term engagement.

### Limitations and lessons learned from applying a citizen science approach

Our study deliberately applied a citizen science approach to investigate pollination services under real-world gardening conditions. This freedom-based protocol introduced substantial environmental and management variability that was not experimentally controlled. While this variability provides valuable opportunities to study ecological interactions within the diversity of actual gardening practices, it complicates the isolation of specific ecological drivers. Although we detected a relationship between landscape imperviousness, bee diversity, and pollination services for *Cucurbitaceae* crops, additional non-pollinator-related factors likely influenced fruit set in both plant families. For example, the lack of a detectable relationship between bee species richness and tomato fruit set may partly reflect variation among tomato cultivars, which likely differed in their pollination requirements and fruiting potential. Combined with environmental and management heterogeneity, this variation may have obscured underlying patterns, as different tomato varieties can show substantial differences in fruit set under drought stress [33]. We assume, however, that gardeners cared for their plants according to their knowledge and with the goal of achieving a successful harvest. Even if specific management instructions such as irrigation schedules had been provided, it is unlikely that identical growing conditions for all study plants could have been ensured across gardens.

At the same time, adapting methods and training to participants’ interests, needs and skills can substantially improve engagement and retention [42]. Future studies could better account for non-pollinator influences on fruit set by systematically collecting additional management data such as soil type, irrigation frequency or plant health status. Encouraging participants to document observations, thoughts and challenges in their own words may further help capture real-world complexity. However, additional tasks designed to increase experimental control may also complicate protocols and potentially reduce participation retention, highlighting an inherent trade-off in citizen science research [12].

Beyond environmental heterogeneity, participant measurement behavior also influenced the collected data. Contrary to our expectations, and despite the long-term commitment of most participants, differences in sampling frequency led to significant differences in fruit set measurements within the *Solanaceae* group. Tomatoes often occur as small-fruited cultivars, which may be more prone to unnoticed removal or loss between observation intervals. This finding suggests that *Solanaceae* crops, particularly tomatoes, may be less suitable focal crops for pollination-focused citizen science studies. Moreover, our instructions did not explicitly address how to adapt observations in the case of interruptions like vacations, even though the flexible design of the protocol helped maintain engagement. In hindsight, clearer and more continuous communication of data quality standards and adaptive procedures would likely have improved data consistency [43].

Data completeness was further affected by quality control requirements and by the flexibility of the protocol. In line with challenges described by Lynch and Miller [48], communication and coordination with participants were affected by the COVID-19 pandemic, resulting in some observations starting only after fruiting had already begun and limiting comparability across gardens. In addition, some datasets had to be excluded from ecological analyses due to insufficient repetitions per garden. Furthermore, allowing participants to freely select study crops reduced sample sizes per crop species, which required combining species in analyses and may have masked crop-specific relationships between explanatory variables and fruit set. Future citizen science pollination studies should therefore aim to ensure sufficient sample sizes per crop species to enable detection of crop-specific pollination patterns. This could be achieved, for example, by assigning specific crops to participants or by coordinating crop selection across study sites. Involving garden coordinators more directly in organizing and monitoring data collection could also improve data completeness while fostering collaboration within gardens.

More generally, although we found no evidence of premature participant drop-out due to commonly reported challenges in citizen science projects, we recommend proactively addressing potential problems during data collection and encouraging participants to continue observations throughout the full plant life cycle, even when plant health declines. Early engagement with the target participant group is essential to understand motivations and interests and to establish clear and consistent communication of data quality criteria. Ideally, study designs should be developed collaboratively with members of the intended participant group [36]. Providing contextual and educational support may further strengthen participation and help address the widespread lack of accessible pollinator education highlighted by Bloom and Crowder [8]. Even citizen science projects that do not depend on taxonomic expertise can contribute to closing this gap by providing concise, accessible training materials.

## Conclusions

Overall, our study underlines the potential of diverse bee communities to mitigate some of the negative effects of urbanization on crop productivity in urban community gardens, at least for insect-pollinated crops with high dependency. In addition, our findings indicate that citizen science can be used to study pollination under real-world gardening conditions, even when levels of participation vary. Future studies could build on these findings by focusing on a smaller set of suitable crop species as well as collecting contextual data in an open-ended way, encouraging participants to document site conditions, management practices, and unexpected influences. This supports ecological interpretation while remaining open to real-world gardening complexity. Building on these insights, we have continued to strengthen our collaboration with gardeners to co-develop interventions for pollinator-friendly gardening [21]. In this sense, community gardens are not only valuable research sites for urban ecology, but also living laboratories for participatory science and community engagement.

## Supplementary Information

Below is the link to the electronic supplementary material.

Supplementary Material 1

## Data Availability

The data that support the findings of this study are openly available in Zenodo at [https://zenodo.org/records/18799227](https:/zenodo.org/records/18799227) . The participants’ comments are not publicly available as the participants provided the data specifically for the purpose of research in the context of the pollinator CS project. However, data on the comments presented in this study are available upon request from the corresponding author.
