# Supplementary material for: Citizen science provides insights on pollination services in urban community gardens
Source: BMC Ecol Evol. 2026 Mar 13;26:26. doi: 10.1186/s12862-026-02507-x (PMC13003671; doi:10.1186/s12862-026-02507-x)
Supplement: Supplementary file 1 — Supplementary Material 1 [file 12862_2026_2507_MOESM1_ESM.docx]

# Supplementary Information

*Table S1* Overview of the participating gardens, participation year, the city they are located and garden characteristics, i.e. garden size, bee metrics and environmental variables. Gardens names in bold indicate data included in the final analysis.

| **Garden** | **Year** | **City** | **Garden size (m^2^)** | **Bee species** | **Bee abundance** | **Temperature (°C)** | **Impervious surface (%)** |
| --- | --- | --- | --- | --- | --- | --- | --- |
| ES | 2021 | Munich | 636.03 | 22 | 338 | 18.37 | 51.43 |
| **GASFK** | 2020 | Berlin | 12612.90 | 17 | 26 | 20.72 | 66.53 |
| **GASTS** | 2020 | Berlin | 23924.91 | 19 | 40 | 20.55 | 32.72 |
| GASTS | 2021 | Berlin | 23924.91 | 15 | 228 | 20.18 | 32.72 |
| **GDB** | 2020 | Berlin | 3843.48 | 29 | 69 | 21.46 | 29.59 |
| **GDB** | 2021 | Berlin | 3843.48 | 27 | 171 | 20.17 | 29.59 |
| GG | 2021 | Munich | 310.80 | 19 | 220 | 19.11 | 60.24 |
| GP | 2021 | Munich | 787.57 | 20 | 169 | 18.92 | 55.53 |
| HB | 2020 | Berlin | 1685.64 | 27 | 82 | 20.70 | 58.45 |
| IGF | 2021 | Munich | 851.22 | 25 | 317 | 19.53 | 67.13 |
| KF | 2020 | Berlin | 8738.35 | 40 | 66 | 20.68 | 23.85 |
| **KF** | 2021 | Berlin | 8738.35 | 19 | 254 | 20.42 | 23.85 |
| **KLG** | 2020 | Berlin | 1560.61 | 27 | 55 | 20.99 | 96.16 |
| **OBZ** | 2021 | Munich | 800.55 | 26 | 315 | 18.40 | 37.20 |
| **PAS** | 2021 | Munich | 351.63 | 10 | 35 | 19.87 | 79.47 |
| PGK | 2021 | Berlin | 1517.00 | 22 | 203 | 19.77 | 20.88 |
| **POL** | 2020 | Berlin | 3900.41 | 39 | 77 | 20.75 | 38.61 |
| POL | 2021 | Berlin | 3900.41 | 29 | 220 | 20.88 | 38.61 |
| PWVK | 2021 | Berlin | 1469.39 | 28 | 203 | 20.84 | 63.42 |
| **RB** | 2020 | Berlin | 557.02 | 18 | 46 | 19.87 | 45.58 |
| **SA** | 2021 | Munich | 1203.02 | 29 | 321 | 19.22 | 49.49 |
| **SCH** | 2020 | Berlin | 432.72 | 11 | 20 | 20.10 | 24.45 |
| SCH | 2021 | Berlin | 432.72 | 12 | 47 | 20.13 | 24.45 |
| **SFM** | 2020 | Berlin | 676.78 | 33 | 72 | 20.29 | 27.82 |
| **SFM** | 2021 | Berlin | 676.78 | 32 | 254 | 20.24 | 27.82 |
| SG | 2021 | Munich | 946.47 | 19 | 579 | 19.25 | 63.95 |
| **SS** | 2021 | Munich | 1433.52 | 27 | 327 | 18.38 | 25.32 |
| **WS** | 2020 | Berlin | 6530.69 | 27 | 45 | 20.17 | 49.43 |

*Table S2* Overview of the observed crops in the final data set and the average fruit set of each crop type.

| **Crop type** | **Number of observations** | **Average fruit set (%)** | **SD** |
| --- | --- | --- | --- |
| Cucumber | 13 | 35.6 | 22.1 |
| Peperoni | 4 | 57.6 | 9.28 |
| Pepper | 3 | 48.1 | 3.21 |
| Pumpkin | 19 | 34.0 | 15.3 |
| Strawberry | 4 | 61.8 | 23.1 |
| Tomato | 35 | 59.0 | 24.5 |
| Zucchini | 15 | 37.9 | 26.7 |
| Total | 93 | 46.9 | 24.4 |

Model structure of the abundance-based LMMs:

1. *Fruit set ~ bee abundance * landscape imperviousness + temperature + garden size + year + (1|garden)*
2. *Fruit set ~ bee abundance * temperature + landscape imperviousness + garden size + year + (1|garden)*

*Table S3* Linear Mixed Models for fruit set of all observations analyzed jointly. Bee richness, bee abundance, imperviousness in a 1000 m buffer, temperature and garden size are scaled values. Colon indicates interaction term. Significant results are in bold.

| **Fixed parameters** | **Estimate** | **SE** | **df** | **t-value** | **Pr(>\|t\|)** |
| --- | --- | --- | --- | --- | --- |
| **Total data set** |  |  |  |  |  |
| **Model 1: richness:imperviousness** |  |  |  |  |  |
| Intercept | 35.414 | 5.520 | 12.667 | 6.416 | <0.001 |
| Bee richness | -4.107 | 4.385 | 7.099 | -0.937 | 0.380 |
| Imperviousness 1000 m | -1.414 | 4.539 | 6.729 | -0.312 | 0.765 |
| Temperature | 0.352 | 3.650 | 13.884 | 0.096 | 0.925 |
| Family (*Solanaceae*) | 24.402 | 4.347 | 79.574 | 5.614 | **<0.001** |
| Garden size | -4.424 | 3.958 | 8.976 | -1.118 | 0.293 |
| Year (2021) | -1.287 | 6.552 | 31.741 | -0.196 | 0.846 |
| Bee richness: Imperviousness | 5.448 | 3.757 | 6.996 | 1.450 | 0.190 |
| **Model 2: richness:temperature** |  |  |  |  |  |
| Intercept | 33.839 | 4.873 | 19.857 | 6.945 | <0.001 |
| Bee richness | -3.146 | 3.423 | 8.898 | -0.919 | 0.382 |
| Imperviousness 1000 m | -2.005 | 3.488 | 7.986 | -0.575 | 0.581 |
| Temperature | -1.324 | 3.147 | 9.094 | -0.421 | 0.684 |
| Family (*Solanaceae*) | 25.192 | 4.352 | 81.585 | 5.789 | **<0.001** |
| Garden size | -1.991 | 3.368 | 12.466 | -0.591 | 0.565 |
| Year (2021) | -2.695 | 6.030 | 37.297 | -0.447 | 0.658 |
| Bee richness: Temperature | 8.821 | 3.744 | 27.769 | 2.356 | **0.026** |
| **Model 3: abundance:imperviousness** |  |  |  |  |  |
| Intercept | 41.425 | 4.424 | 16.886 | 9.364 | <0.001 |
| Bee abundance | -8.772 | 3.062 | 16.047 | -2.865 | **0.011** |
| Imperviousness 1000 m | -3.809 | 3.290 | 8.867 | -1.158 | 0.277 |
| Temperature | 0.582 | 2.722 | 13.008 | 0.214 | 0.834 |
| Family (*Solanaceae*) | 24.592 | 4.192 | 83.653 | 5.867 | **<0.001** |
| Garden size | -6.214 | 2.960 | 13.113 | -2.099 | 0.056 |
| Year (2021) | -10.790 | 5.568 | 16.424 | -1.938 | 0.070 |
| Bee abundance: Imperviousness | 6.680 | 2.506 | 16.791 | 2.666 | **0.016** |
| **Model 4: abundance:temperature** |  |  |  |  |  |
| Intercept | 36.691 | 5.825 | 11.028 | 6.299 | <0.001 |
| Bee abundance | -7.923 | 3.646 | 9.825 | -2.173 | 0.055 |
| Imperviousness 1000 m | -5.809 | 4.477 | 6.717 | -1.297 | 0.237 |
| Temperature | -1.031 | 4.276 | 6.077 | -0.241 | 0.817 |
| Family (*Solanaceae*) | 25.670 | 4.288 | 81.824 | 5.987 | **<0.001** |
| Garden size | -5.160 | 3.868 | 8.839 | -1.334 | 0.216 |
| Year (2021) | -7.525 | 9.110 | 12.552 | -0.826 | 0.424 |
| Bee abundance: Temperature | 2.287 | 5.433 | 17.494 | 0.421 | 0.679 |

*Table S4* Linear Mixed Models for fruit set of *Cucurbitaceae.* Bee richness, bee abundance, imperviousness in a 1000 m buffer, temperature and garden size are scaled values. Colon indicates interaction term. Significant results are in bold.

| **Fixed parameters** | **Estimate** | **SE** | **df** | **t-value** | **Pr(>\|t\|)** |
| --- | --- | --- | --- | --- | --- |
| ***Cucurbitaceae*** |  |  |  |  |  |
| **Model 5: richness:imperviousness** |  |  |  |  |  |
| Intercept | 43.455 | 4.102 | 40.000 | 10.595 | <0.001 |
| Bee richness | -3.102 | 3.189 | 40.000 | -0.973 | 0.337 |
| Imperviousness 1000 m | -1.065 | 3.486 | 40.000 | -0.305 | 0.762 |
| Temperature | -1.007 | 3.039 | 40.000 | -0.331 | 0.742 |
| Garden size | -4.799 | 3.001 | 40.000 | -1.599 | 0.118 |
| Year (2021) | -11.48 | 6.229 | 40.000 | -1.843 | 0.073 |
| Bee richness: Imperviousness | 5.717 | 2.694 | 40.000 | 2.122 | **0.040** |
| **Model 6: richness:temperature** |  |  |  |  |  |
| Intercept | 39.321 | 4.829 | 40.000 | 8.142 | <0.001 |
| Bee richness | -0.861 | 3.075 | 40.000 | -0.280 | 0.781 |
| Imperviousness 1000 m | -1.828 | 3.561 | 40.000 | -0.513 | 0.610 |
| Temperature | -2.937 | 3.004 | 40.000 | -0.978 | 0.334 |
| Garden size | -1.797 | 3.221 | 40.000 | -0.558 | 0.580 |
| Year (2021) | -9.012 | 7.284 | 40.000 | -1.237 | 0.223 |
| Bee richness: Temperature | 6.682 | 4.243 | 40.000 | 1.575 | 0.123 |
| **Model 7: abundance:imperviousness** |  |  |  |  |  |
| Intercept | 44.127 | 3.993 | 40.000 | 11.052 | <0.001 |
| Bee abundance | -5.197 | 3.057 | 40.000 | -1.700 | 0.097 |
| Imperviousness 1000 m | -1.254 | 3.437 | 40.000 | -0.365 | 0.717 |
| Temperature | -2.026 | 2.786 | 40.000 | -0.727 | 0.471 |
| Garden size | -4.519 | 2.855 | 40.000 | -1.583 | 0.121 |
| Year (2021) | -12.252 | 6.001 | 40.000 | -2.042 | **0.048** |
| Bee abundance: Imperviousness | 6.386 | 2.539 | 40.000 | 2.515 | **0.016** |
| **Model 8: abundance:temperature** |  |  |  |  |  |
| Intercept | 41.168 | 5.216 | 2.826 | 7.893 | 0.005 |
| Bee abundance | -2.697 | 3.268 | 3.874 | -0.825 | 0.457 |
| Imperviousness 1000 m | -3.860 | 4.073 | 2.623 | -0.948 | 0.422 |
| Temperature | -3.686 | 3.549 | 1.987 | -1.038 | 0.409 |
| Garden size | -3.337 | 3.429 | 3.517 | -0.973 | 0.393 |
| Year (2021) | -11.195 | 9.281 | 4.424 | -1.206 | 0.288 |
| Bee abundance: Temperature | 3.077 | 5.407 | 4.248 | 0.569 | 0.598 |

*Table S5* Linear Mixed Models for fruit set of *Solanaceae* including strawberry. Bee richness, bee abundance, imperviousness in a 1000 m buffer, temperature and garden size are scaled values. Colon indicates interaction term. Significant results are in bold.

| **Fixed parameters** | **Estimate** | **SE** | **df** | **t-value** | **Pr(>\|t\|)** |
| --- | --- | --- | --- | --- | --- |
| ***Solanaceae*** |  |  |  |  |  |
| **Model 9: richness:imperviousness** |  |  |  |  |  |
| Intercept | 60.264 | 7.246 | 8.875 | 8.316 | <0.001 |
| Bee richness | -10.614 | 8.509 | 5.440 | -1.247 | 0.263 |
| Imperviousness 1000 m | -3.060 | 5.655 | 5.448 | -0.541 | 0.610 |
| Temperature | 5.461 | 4.875 | 9.723 | 1.120 | 0.289 |
| Garden size | -7.827 | 6.406 | 11.415 | -1.222 | 0.246 |
| Year (2021) | -2.169 | 9.149 | 24.268 | -0.237 | 0.815 |
| Bee richness: Imperviousness | -10.189 | 9.542 | 4.469 | -1.068 | 0.340 |
| **Model 10: richness:temperature** |  |  |  |  |  |
| Intercept | 58.299 | 7.128 | 13.019 | 8.179 | <0.001 |
| Bee richness | -5.817 | 7.119 | 9.331 | -0.817 | 0.434 |
| Imperviousness 1000 m | -1.983 | 5.577 | 7.507 | -0.356 | 0.732 |
| Temperature | 1.862 | 5.395 | 8.485 | 0.345 | 0.738 |
| Garden size | -3.351 | 6.191 | 15.144 | -0.541 | 0.596 |
| Year (2021) | -1.175 | 9.076 | 29.619 | -0.129 | 0.898 |
| Bee richness: Temperature | 8.747 | 6.691 | 19.215 | 1.307 | 0.207 |
| **Model 11: abundance:imperviousness** |  |  |  |  |  |
| Intercept | 65.910 | 6.567 | 10.645 | 10.037 | <0.001 |
| Bee abundance | -15.810 | 6.074 | 10.811 | -2.603 | **0.025** |
| Imperviousness 1000 m | -6.054 | 4.846 | 7.666 | -1.249 | 0.248 |
| Temperature | 4.353 | 4.128 | 12.184 | 1.054 | 0.312 |
| Garden size | -10.793 | 5.467 | 14.924 | -1.974 | 0.067 |
| Year (2021) | -10.750 | 8.839 | 14.703 | -1.216 | 0.243 |
| Bee abundance: Imperviousness | -1.279 | 6.992 | 8.128 | -0.183 | 0.859 |
| **Model 12: abundance:temperature** |  |  |  |  |  |
| Intercept | 66.992 | 6.801 | 8.676 | 9.850 | <0.001 |
| Bee abundance | -15.587 | 5.579 | 18.504 | -2.794 | **0.012** |
| Imperviousness 1000 m | -6.635 | 5.062 | 7.151 | -1.311 | 0.231 |
| Temperature | 5.486 | 5.423 | 8.254 | 1.012 | 0.341 |
| Garden size | -11.167 | 5.380 | 14.369 | -2.076 | 0.056 |
| Year (2021) | -13.197 | 10.880 | 11.792 | -1.213 | 0.249 |
| Bee abundance: Temperature | -2.438 | 7.548 | 22.174 | -0.323 | 0.750 |

*Table S6* Comparison of mixed-effects models explaining fruit set using interactions between bee metrics and environmental variables for the total dataset and for *Cucurbitaceae* and *Solanaceae* separately. Model fit is evaluated using AIC, AICc, marginal R² and conditional R².

| **Data set** | **Model** | **Interaction** | **AIC** | **AICc** | **Marg. R^2^** | **Cond. R^2^** |
| --- | --- | --- | --- | --- | --- | --- |
| Total | 1 | Richness × Imperviousness | 806.85 | 809.12 | 0.2666 | 0.4864 |
|  | 2 | Richness × Temperature | 804.12 | 806.29 | 0.3027 | 0.4129 |
|  | 3 | Abundance × Imperviousness | 799.16 | 801.33 | 0.3684 | 0.4440 |
|  | 4 | Abundance × Temperature | 803.89 | 806.05 | 0.2827 | 0.4446 |
| *Cucurbitaceae* | 5 | Richness × Imperviousness | 392.49 | 396.28 | 0.2585 | 0.2585 |
|  | 6 | Richness × Temperature | 393.49 | 397.28 | 0.2268 | 0.2268 |
|  | 7 | Abundance × Imperviousness | 390.34 | 394.13 | 0.2952 | 0.2952 |
|  | 8 | Abundance × Temperature | 394.56 | 398.35 | 0.1874 | 0.2211 |
| *Solanaceae* | 9 | Richness × Imperviousness | 387.28 | 391.17 | 0.1015 | 0.4400 |
|  | 10 | Richness × Temperature | 387.45 | 391.34 | 0.0726 | 0.4128 |
|  | 11 | Abundance × Imperviousness | 383.47 | 387.36 | 0.2164 | 0.4189 |
|  | 12 | Abundance × Temperature | 383.21 | 387.10 | 0.2271 | 0.4094 |

*Table S7* Share of contributions (i.e., study plants examined) per participation type that included a comment on plant health, plant death, harvest loss and the protocol in % for the crop group *Solanaceae* including strawberry (n = 83). Note: A contribution may contain comments relating to several categories, which means that the total percentage of all categories may exceed 100 %.

| **Participation type** | **Plant health** | **Plant death** | **Harvest loss** | **Protocol** |
| --- | --- | --- | --- | --- |
| Short duration,  low frequency | 31 | 15 | 31 | 15 |
| Short duration,  high frequency | 38 | 0 | 25 | 13 |
| Long duration,  low frequency | 37 | 7 | 43 | 13 |
| Long duration,  high frequency | 19 | 0 | 32 | 35 |

*Table S8* Share of contributions (i.e., study plants examined) per participation type that included a comment on plant health, plant death, harvest loss and the protocol in % for the crop group *Cucurbitaceae* (n = 65). Note: A contribution may contain comments relating to several categories, which means that the total percentage of all categories may exceed 100 %.

| **Participation type** | **Plant health** | **Plant death** | **Harvest loss** | **Protocol** |
| --- | --- | --- | --- | --- |
| Short duration,  low frequency | 29 | 43 | 0 | 14 |
| Short duration,  high frequency | 29 | 0 | 48 | 14 |
| Long duration,  low frequency | 50 | 15 | 25 | 30 |
| Long duration,  high frequency | 48 | 4 | 30 | 37 |
